# Supplementary material for: Topological digestion drives time-varying rheology of entangled DNA fluids
Source: Nat Commun. 2022 Jul 28;13:4389. doi: 10.1038/s41467-022-31828-w (PMC9334285; doi:10.1038/s41467-022-31828-w)
Supplement: Supplementary file 1 — Supplementary Information [file 41467_2022_31828_MOESM1_ESM.pdf]

Topological digestion drives time-varying rheology of entangled DNA fluids

D. Michieletto,[1,2,*] P. Neill,[3] S. Weir,[3] D. Evans,[1] N. Crist,[3] V. A. Martinez,[1] and R. M. Robertson-Anderson[3,†]

[1]*School of Physics and Astronomy, University of Edinburgh, Peter Guthrie Road, Edinburgh, EH9 3FD, UK*
[2]*MRC Human Genetics Unit, Institute of Genetics and Cancer, University of Edinburgh, Edinburgh EH4 2XU, UK*
[3]*Department of Physics and Biophysics, University of San Diego, 5998 Alcala Park, San Diego, CA, 92110*

Supplementary Figures

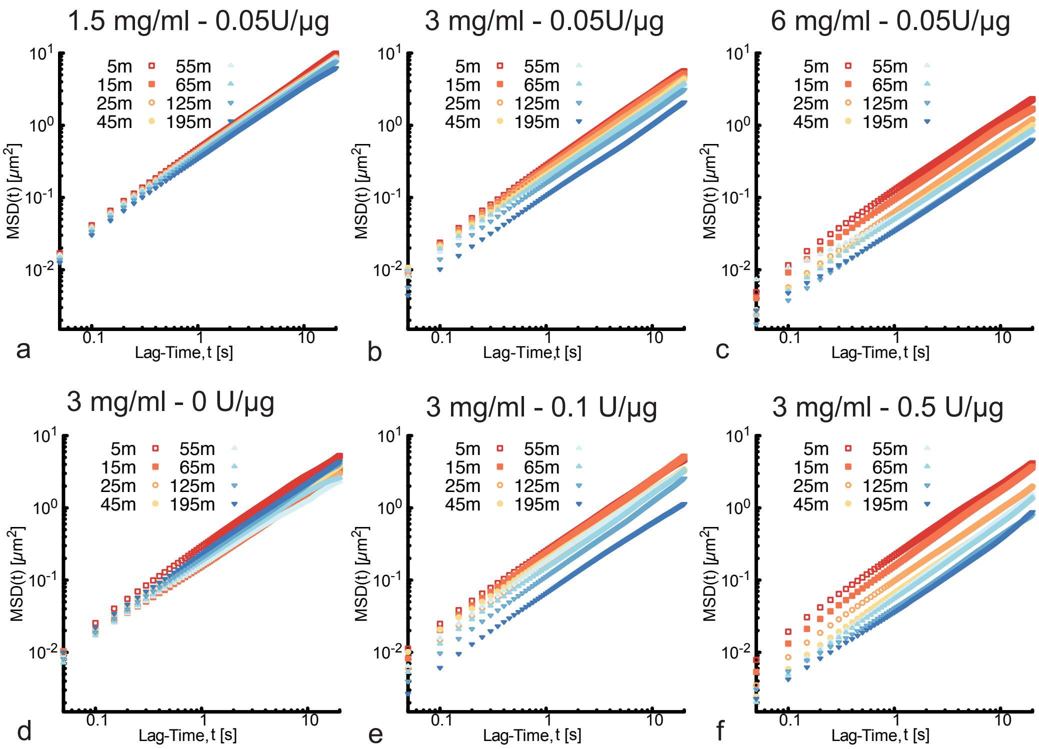

Supplementary Figure 1: *MSD*s for solutions of pYES2 DNA undergoing linearization by BamHI-HF at (top row) different DNA concentrations and fixed stoichiometry and (bottom row) different stoichiometry (including 0 U/μg) and fixed DNA concentration.

---

*davide.michieletto@ed.ac.uk
†randerson@sandiego.edu

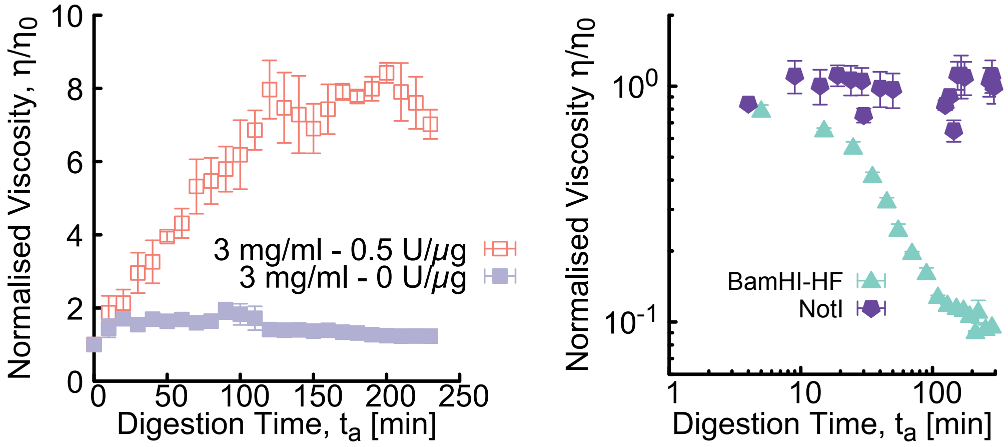

Supplementary Figure 2: **Left.** Normalised viscosity of pYES2 plasmids undergoing digestion with BamHI at 0 U/μg and 0.5 U/μg. **Right.** Normalised viscosity of entangled λ-DNA undergoing digestion by BamHI-HF (5 cleaving sites) compared to an RE that does not cut λ-DNA (NotI).

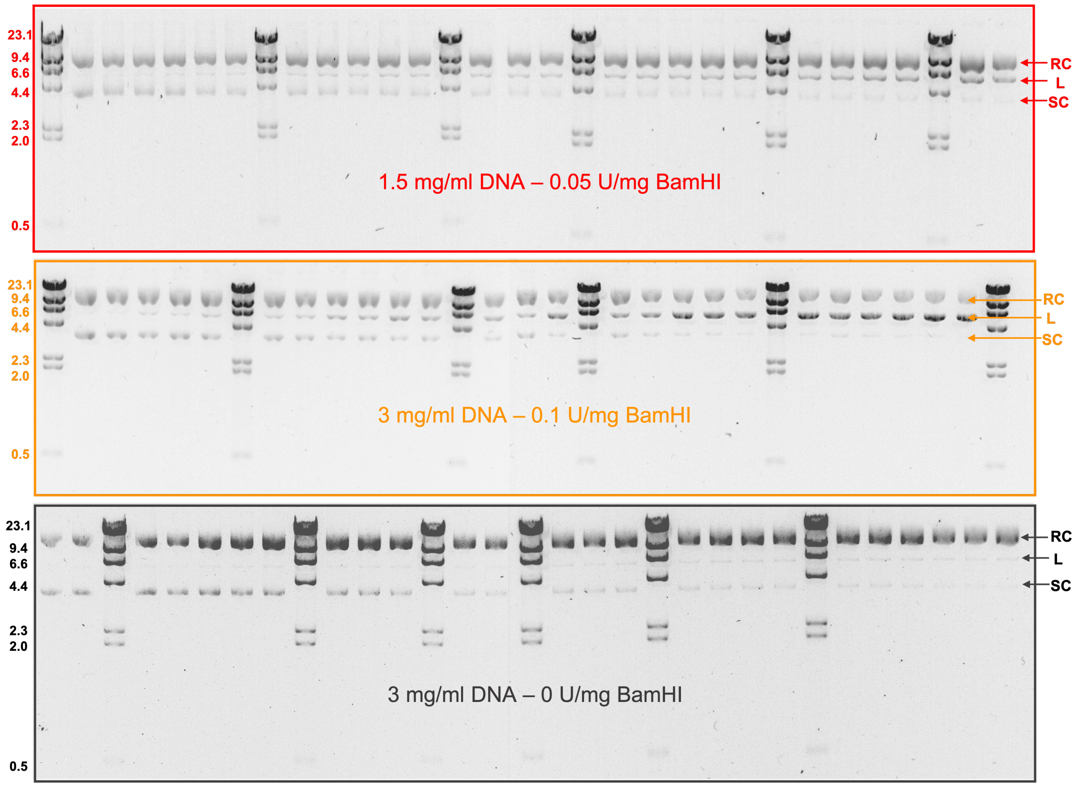

Supplementary Figure 3: Gel electrophoresis of pYES2 DNA undergoing digestion by BamHI. In each gel the different lanes show the different DNA topologies (L = linear, SC = supercoiled, R = relaxed circular (ring)) at varying time-points t_a during the digestion starting at 0 min and proceeding in 10-minute intervals for 240 minutes. The DNA concentration and stoichiometry are listed on the corresponding gel and color-coded according to figures in the main text. Marker lanes (those with 7 bands) are the standard λ-HindIII ladder with the length of each linear strand listed in kbp to the left of the gel. The other concentrations and stoichiometries tested are shown in Fig. 1 in the main text.

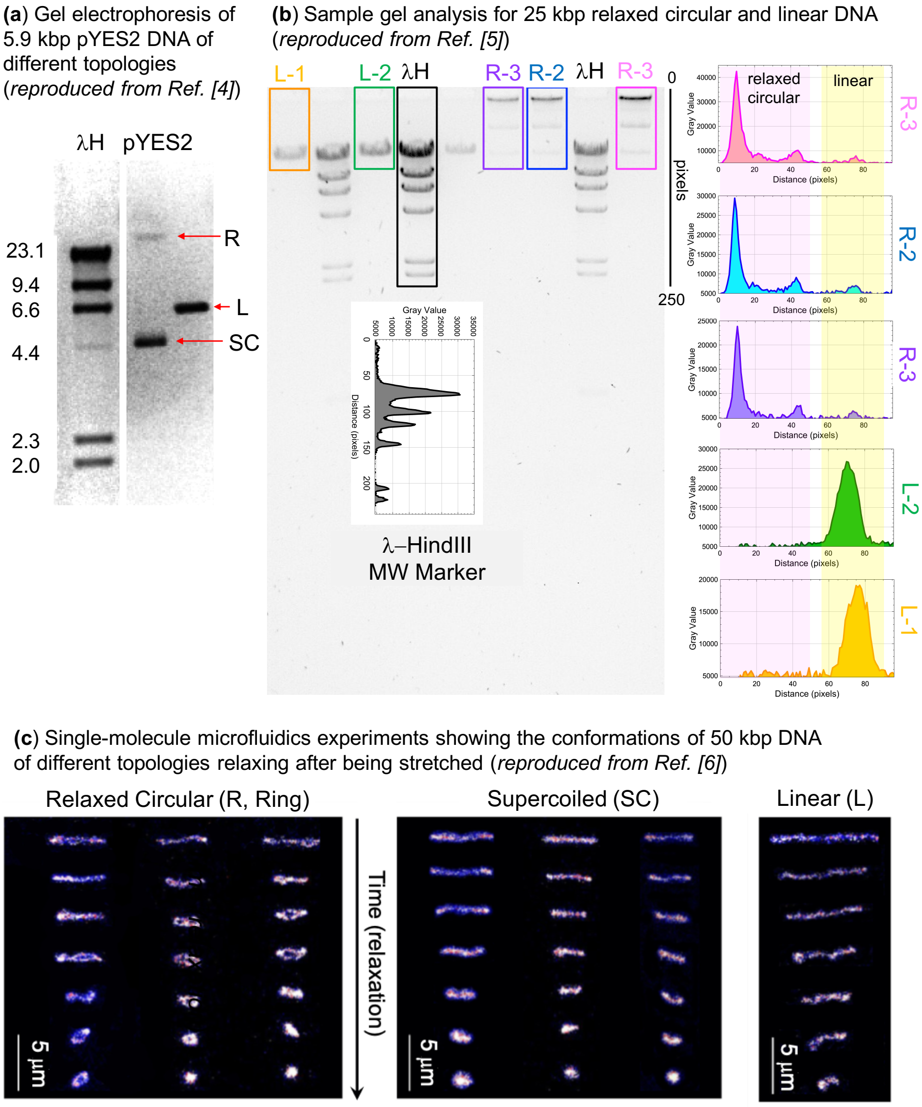

Supplementary Figure 4: **Analyzing DNA topology using gel electrophoresis (a), band intensity analysis (b) and single-molecule imaging (c).** **(a)** Agarose gel electrophoresis of pYES2 DNA used in experiments shown in Figs 1 and 3. The first lane corresponds to the standard λ-HindIII (λH) molecular weight marker with the lengths of linear bands listed in kbp while the 2nd and 3rd lanes correspond to supercoiled (SC), ring (R) and Linear topologies as indicated. Gel image is reproduced from Ref. [1]. **(b)** Agarose gel electrophoresis of 25 kbp linear (L) and ring (R) DNA used to assess the topology and concentration of the DNA. L-1 (orange) and L-2 (green) are lanes containing different dilutions of a linear DNA solution. RC-1 (purple), RC-2 (blue) and RC-3 (magenta) contain different dilutions of a mostly circular DNA solution. The histograms to the right of the gel show the intensity histogram along a vertical line through the center of each lane. To determine the relative concentrations of ring and linear DNA in each sample we analyze the relative area of the intensity distribution corresponding to each band using Life Technologies E-Gel Imager and Gel Quant Express software. The peaks centered at $\sim$10 pixels, $\sim$45 pixels, and $\sim$75 pixels correspond to relaxed circular (ring), supercoiled and linear topology. Linear DNA with length $>$20 kbp aligns with the first band in the marker, while ring DNA runs more slowly through the gel and often get permanently arrested in the loading well. Image is reproduced from Ref. [2]. **(c)** Single-molecule snapshots of 50 kbp relaxed ring (left), supercoiled (middle) and linear (right) DNA molecules within the same sample during the relaxation phase after stretching under planer extensional flow. 231 molecules in total were stretched and imaged during relaxation to determine the fraction of ring, supercoiled and linear molecules in the blend. The fractions determined from single-molecule flow experiments and gel electrophoresis analysis were within $\lesssim 3\%$ of each other. Images are reproduced from Ref. [3].

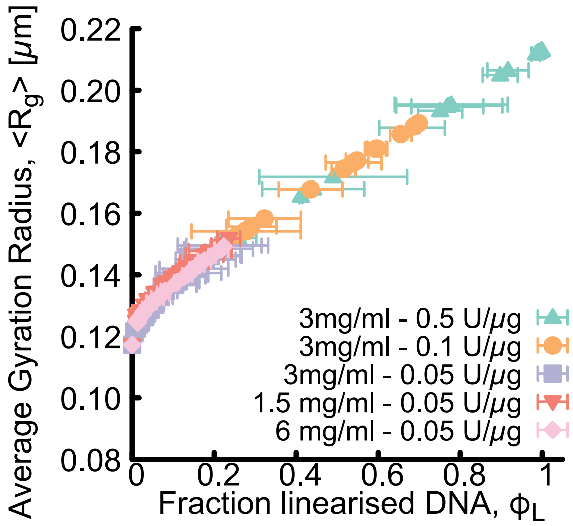

Supplementary Figure 5: Average radius of gyration $\langle R_g \rangle$ of pYES2 DNA undergoing digestion by BamHI plotted against fraction of linearised DNA ϕ_L. Both $\langle R_g \rangle$ and ϕ_L are determined from time-resolved quantitative gel electrophoresis as described in Methods and SI Figs S3,S4.

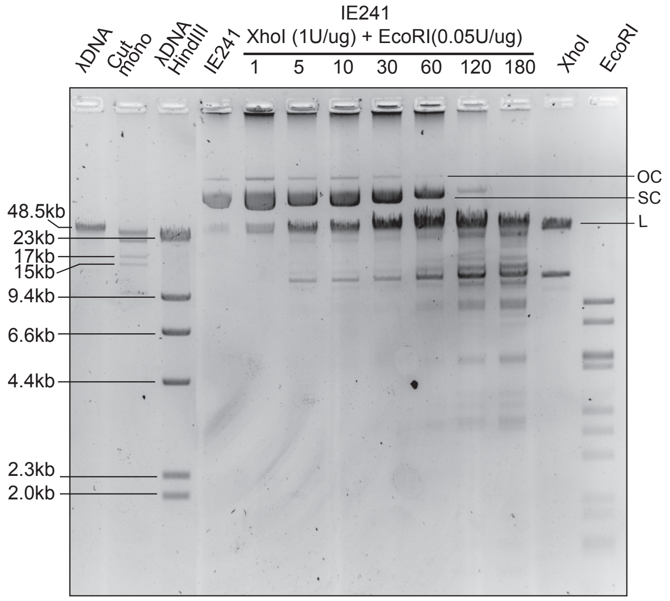

Supplementary Figure 6: Gel electrophoresis of IE241 plasmids undergoing digestion by a combination of XhoI (cuts the plasmid twice) and EcoRI (cuts the plasmid 10 times). The digestion time of each kinetic aliquot is listed above the corresponding lane. The last two lanes show fully digested IE241 plasmid by XhoI and EcoRI only. OC = open circular (or ring). SC = supercoiled. L = linear.

Supplementary Table 1.

RE	Site	Ends	λ-DNA	PYES2	IE241
HindIII	AAGCTT	overhang	6	-	2
BamHI	GGATCC	overhang	5	1	9
EcoRI	GAATTC	overhang	5	-	10
ScaI	AGTACT	blunt	5	-	2
HaeIII	GGCC	blunt	141	-	-
NotI	GCGGCCGC	overhang	0	-	-
XhoI	CTCGAG	overhang	1	-	2

Supplementary Table I: Table of the restriction enzymes used in this work. Columns are as follows: RE = name of restriction enzyme, Site = DNA basepair string in RE recognition site, Ends = type of cleaving the RE performs (leaving single-strand overhangs that are sticky or blunt ends that are not), λ-DNA = number of recognition sites on λ-DNA ; PYES2 = number of recognition sites on PYES2; IE241 = number of recognition sites on IE241; Dashes indicate that the combination was not tested.

Supplementary Note 1.

Brownian Dynamics Simulations

DNA is modelled as a twistable elastic chain (as detailed in Ref. [4]) whereby the backbone is made of beads each decorated by three non-interacting beads (referred to as "patches"). The backbone bead and the 3 patches behave as a unique rigid body. The backbone beads interact via a purely repulsive Lennard-Jones potential as

$$U_{\rm LJ}(r) = \begin{cases} 4\epsilon\left[\left(\frac{\sigma_b}{r}\right)^{12} - \left(\frac{\sigma_b}{r}\right)^{6} + \frac{1}{4}\right] & r \le r_c \\ 0 & r > r_c \end{cases}, \tag{1}$$

where r denotes the separation between the bead centers. The cutoff distance $r_c = 2^{1/6}\sigma$ is chosen so that only the repulsive part of the Lennard-Jones is used. The energy scale is set by $\epsilon = \kappa_B T$ and the length scale by σ_b, both of which are set to unity in our simulations. Consistent with this, all quantities are reported in reduced LJ units. The size of each bead in real units should be considered as the thickness of DNA, i.e. $\sigma = 2.5$ nm or $= 2.5/0.34 \simeq 7.35$ bp per bead. The patches have no steric interactions.

Nearest-neighbour beads along the backbone are connected by finitely extensible nonlinear elastic (FENE) springs as

$$U_{\rm FENE}(r) = \begin{cases} -0.5kR_0^2 \ln\left(1-(r/R_0)^2\right) & r \le R_0 \\ \infty & r > R_0 \end{cases}, \tag{2}$$

where $k = 30\epsilon/\sigma_b^2$ is the spring constant and $R_0 = 1.5\sigma_b$ is the maximum extension of the elastic FENE bond.

To model the persistence length of DNA (150 bp or 50 nm) we introduce an additional bending energy penalty between consecutive triplets of neighbouring beads along the backbone in order to control polymer stiffness:

$$U_{\rm bend}(\theta_b) = k_\theta\left(1+\cos\theta_b\right) . \tag{3}$$

Here, θ_b is the angle formed between adjacent bonds, i.e. $\boldsymbol{t}_i \cdot \boldsymbol{t}_{i+1}/|\boldsymbol{t}_i||\boldsymbol{t}_{i+1}|$ with $\boldsymbol{t}_i$ the tangent at i, and $k_\theta = 20\kappa_B T$ is the bending constant. With this choice $l_p = 20\sigma_b \simeq 50$ nm is the persistence length.

To model the torsional stiffness, two dihedral CHARMM springs constrain the relative rotation of consecutive beads, ψ, at a user-defined value (ψ_0). The torsional angle ψ is determined as the angle between planes defined by the triplets bead-bead-patch running along the DNA backbone. The potential is

$$U_{\rm torsion}(\psi) = k_\psi\left[1+\cos\left(n\psi - d\right)\right] \tag{4}$$

where $k_\psi = 50k_BT$, $n = 1$ and $d = \psi_0$. The angle ψ_0 directly determines the thermodynamically preferred pitch of the twisted ribbon as $p = 2\pi/\psi_0$ and, in turn, this determines the preferred linking number as $Lk = M/p$, where M is the number of beads in the plasmid. In this model, we define the supercoiling as $\sigma \equiv Lk/M = 1/p$, which is set by initialising the patchy-polymer as a flat ribbon and by subsequently imposing the angle ψ_0 in order to achieve the desired σ (which may be zero, if $\psi_0 = 0$ or $p = \infty$).

Finally, to maintain consecutive beads parallel to the backbone, we constrain the angle between the triplets bead-bead-patch to $\pi/2$ so that the frames of reference formed by the triplets are aligned to each other. This potential is written as

$$U_{\rm align} = k_a\left(1+\cos\theta\right) \tag{5}$$

where θ is the tilt angle with $k_a = 200k_BT$ (see also Ref. [5]).

The simulations are performed at fixed monomer density $\rho\sigma_b^3 = 0.08$ and $\rho\sigma_b^3 = 0.006$, equivalent to ∼39 mg/ml and 3 mg/ml of DNA ($\sigma_b = 2.5$ nm= 7.35 bp is the typical size of a bead). We evolve the equations of motion for the beads coupled to a heath bath which provides noise and friction. The equation of motion for each Cartesian component is thus given by

$$m_a \partial_{tt} r_a = -\nabla U_a - \gamma_a \partial_t r_a + \sqrt{2k_BT\gamma_a}\,\eta_a(t)\,, \tag{6}$$

where m_a and γ_a are the mass and the friction coefficient of bead a, and η_a is its stochastic noise vector satisfying the fluctuation-dissipation theorem. U is the sum of the energy fields described above. The simulations are performed in LAMMPS with $m = \gamma = k_B = T = 1$ and using a velocity-Verlet algorithm with integration time step $\Delta t = 0.002\,\tau_B$, where $\tau_B = \gamma\sigma^2/k_BT \simeq 0.03$ μs (using $\gamma = 3\pi\eta_{water}\sigma$ with $\eta_{water} = 1$ cP and $\sigma = 2.5$ nm) is the Brownian time.

The damp parameter is set to 4, so that the rigid body made of a backbone bead with with 3 patches (all with mass 1) has the same inertial time as a simple bead of mass 1.

To mimic different stages of DNA digestion by restriction enzymes with single restriction sites we remove a single bead from a different fraction f of rings together with the angles and dihedrals in which it is involved. Subsequently, we set the dihedral constant of all the dihedrals belonging to the fraction f of rings to $k_\psi = 0 k_B T$ mimicking fully relaxed linear segments of DNA. This approach is justified by the fact that twist diffuses much faster than writhe in DNA [6] and so a short time after cleavage by a restriction enzyme, supercoiled DNA is likely mostly torsionally relaxed and yet retains much of the unresolved writhe.

Supplementary References

[1] S. Laib, R. M. Robertson, and D. E. Smith, Macromolecules **39**, 4115 (2006), ISSN 0024-9297, URL `https://doi.org/10.1021/ma0601464`.

[2] P. Khanal, K. R. Peddireddy, J. Marfai, R. McGorty, and R. M. Robertson-Anderson, Journal of Rheology **66**, 699 (2022), ISSN 0148-6055.

[3] K. R. Peddireddy, M. Lee, Y. Zhou, S. Adalbert, S. Anderson, C. M. Schroeder, and R. M. Robertson-Anderson, Soft Matter **16**, 152 (2019), ISSN 17446848.

[4] C. A. Brackley, A. N. Morozov, and D. Marenduzzo, J. Chem. Phys. **140**, 135103 (2014), ISSN 1089-7690, URL `http://www.ncbi.nlm.nih.gov/pubmed/24712817`.

[5] J. Smrek, J. Garamella, R. Robertson-Anderson, and D. Michieletto, Science Advances **7**, 1 (2021), ISSN 23752548.

[6] Y. A. G. Fosado, D. Michieletto, C. A. Brackley, and D. Marenduzzo, Proceedings of the National Academy of Sciences **118** (2021).
