# Supplementary figures and images for: Topological digestion drives time-varying rheology of entangled DNA fluids
Source: Nat Commun. 2022 Jul 28;13:4389. doi: 10.1038/s41467-022-31828-w (PMC9334285; doi:10.1038/s41467-022-31828-w)

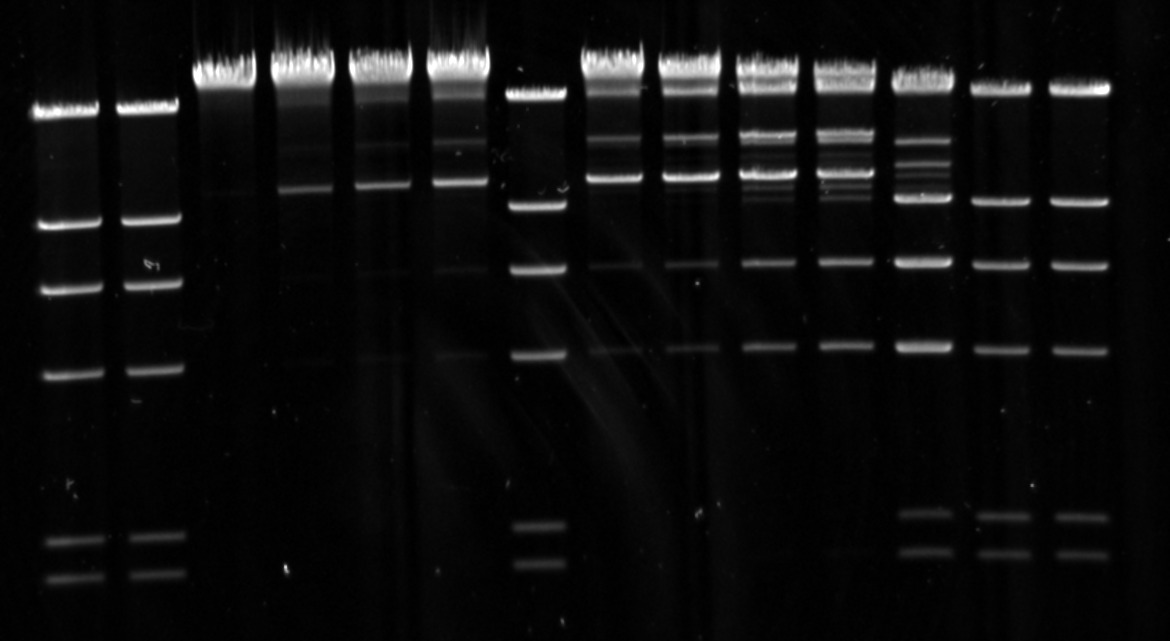

Supplement: Supplementary file 3 — Source Data [file 41467_2022_31828_MOESM3_ESM.zip › SOURCE_DATA/DATA_FIG4/panel_j/LambdaDNA_HindIII.tif]
